# The Relationship Between Leptin and Norepinephrine Levels During OGTT in Normotensive and Hypertensive Obese Adolescents

**DOI:** 10.4274/jcrpe.v1i4.57

**Published:** 2010-12-08

**Authors:** Ping Zhou, Raphael David, Bina Shah

**Affiliations:** 1 Division of Pediatric Endocrinology, School of Medicine, New York University, NY, USA; 2 Division of Pediatric Endocrinology, Albert Einstein College of Medicine, Bronx, NY, USA; +718-920-4664+718-405-5609pzhou@montefiore.orgDivision of Pediatric Endocrinology, Albert Einstein College of Medicine, 3450 Wayne Ave., Bronx, NY 10467

**Keywords:** obesity, hypertension, leptin, norepinephrine, sympathetic nervous system

## Abstract

**Background/Aims**: Studies in adult population have suggested that leptin might play a role in inducing obesity related hypertension mediated by the sympathetic nervous system. This association has not been established for adolescents. Our study is designed to explore the relationship between leptin and norepinephrine levels in pediatric patients and to identify any contributors to hypertension for this population.

**Methods**: Thirty-nine obese adolescents, divided into four groups by gender and hypertension status were included in the study. Leptin and norepinephrine levels were measured during oral glucose tolerance tests (OGTT) to optimize hormonal secretion. T tests were used to compare baseline levels of glucose, insulin, leptin and norepinephrine at 0 hour point of OGTT between the hypertensive and normotensive patients for both genders. Analysis of covariance (ANCOVA) was used for comparison of subsequent levels between the hypertensive and normotensive groups for in both genders, with the corresponding baseline level as the covariance. Models with and without BMI adjustment were created and their results were found to be consistent. Correlation between leptin and norepinephrine was examined at each time point and through analysis of area under the curve (AUC).

**Results**: Contrary to the previous findings obtained in adult patients, our results did not show any direct relationships between levels of leptin and norepinephrine. A slight decrease in norepinephrine level at 1 hour in the normotensive male group and a significant increase in leptin level at 1 hour in the hypertensive female group was observed.

**Conclusion**: Our preliminary data suggest that norepinephrine and leptin levels at 0 and 1 hour during routine OGTT, for males and females, respectively, may help identify a subgroup of obese adolescents who have higher risk for hypertension and cardiovascular complications.

**Conflict of interest:**None declared.

## INTRODUCTION

It has been proposed that leptin, the most abundant adipose-specific protein, plays a central role in inducing hypertension. Animal studies suggest that there is a dual interaction of leptin and the sympathetic nervous system. It is hypothesized that leptin, acting within the hypothalamus, causes activation of central sympathetic outflow and stimulates adrenal medullary release of epinephrine, and conversely, the sympathetic nervous system inhibits leptin release from white adipose tissue.^1-6^ A few studies in adults ^7, 8^ provided some preliminary evidence which also suggested that hyperlepitinemia associated with obesity causes sympathetic over-activity and hypertension. One study found that glucose loading increased circulating leptin concentrations in obese women and demonstrated the existence of an association between leptin and norepinephrine changes during oral glucose tolerance test (OGTT) in both normotensive and hypertensive obese women.^8^

An increment in leptin levels is observed as body fat mass increases. Also, the serum leptin level can change independently of fat mass. Leptin levels decrease during fasting and increase after meals.^9^ Both insulin and glucose have been implicated in modulating serum leptin levels.^10, 11^ In an attempt to assess the maximum effect of leptin on the sympathetic nervous system, we measured the hormonal levels (leptin and norepinephrine) during OGTT.

The goal of this study was to assess the relationship of leptin and norepinepherine in normotensive and hypertensive obese adolescents during an oral glucose challenge test.

## SUBJECTS AND METHODS

This research was conducted in the Clinical Research Center at the New York University (approved by the Internal Review Board of the School of Medicine at NYU). A total of 39 obese adolescents were divided into 4 groups: Group 1: 13 normotensive females; Group 2: 8 hypertensive females; Group 3: 8 normotensive males; and Group 4: 10 hypertensive males. The only inclusion criterion was obesity, regardless of gender, ethnicity, or status of hypertension. Exclusion criteria included any underlying diseases that could have caused hypertension, type 2 diabetes mellitus, or conditions that were being treated with medications.

Obesity was defined as a body mass index (BMI) equal to or greater than 95^th^ percentile for age and sex. Hypertension is generally defined as either a systolic or a diastolic blood pressure that is equal to or greater than the 90^th^ percentile for age, gender, and height.^12^ In our hypertensive groups, both systolic and diastolic blood pressures were above the 90^th^ percentile. All subjects underwent a thorough clinical screening, which included history and physical examination. A comprehensive laboratory assessment included measurement of thyroid hormone, electrolytes, liver functions and renal function tests. We also measured fasting lipid levels.

A 3-hour oral glucose tolerance test was performed on all subjects, which started between 8 to 8.30 am. The subjects had been instructed to fast for at least 10 hours prior to the test. Glucose, insulin, leptin and norepinephrine levels were measured at 0, 1, 2, and 3 hours, respectively. Norepinephrine, leptin and all related tests were analyzed by an independent laboratory (Quest Diagnostics). Norepinephrine was measured by high performance liquid chromatography with both intra-assay and inter-assay variation of less than 10%. Leptin was determined by radioimmunoassay with intra-assay and inter-assay variation of 4.2% and 9.8%, respectively.

**Statistical Analysis**

Areas under the curve (AUC) for leptin, norepinephrine, insulin, and glucose levels were calculated by the trapezoidal rule. Relationships among variables as well as their AUCs were evaluated using the Pearson correlation coefficient and multiple linear regression analysis. Mean levels of leptin, norepinephrine, insulin, and glucose at baseline (0 hour) were compared by gender between the hypertensive group and normotensive group using t tests. Mean levels of leptin, norepinephrine, insulin, and glucose at 1, 2, and 3 hours were compared using analysis of covariance (ANCOVA), between the hypertensive group and normotensive group for both sexes, where the corresponding baseline levels are treated as covariance. Models with and without BMI adjustments were compared. Data were expressed as mean + SE. Two-tailed values of p < 0.05 were considered statistically significant. These analyses were performed using the SAS statistical package (SAS Institute, Cary, NC).

## RESULTS

Neither multiple linear regressions nor correlation analyses showed any direct relationship between leptin and norepinephrine in any of the groups. When t tests were used to compare baseline levels of leptin, norepinephrine, insulin, and glucose between the hypertensive patients and the normotensive patients, no significant differences were found among the groups in either gender.

In ANCOVA comparisons of subsequent levels of leptin, norepinephrine, insulin, and glucose, there was a statistically significant difference between the two male groups in norepinephrine levels at 1 hour during OGTT, the hypertensive male group having a higher mean norepinephrine level compared to the normotensive group (Δ=71.2, and p=0.002 with adjustment for BMI; and Δ=74.7, and p=0.001 without adjustment for BMI).

The only statistically significant difference between the two female groups was in the leptin levels at 1 hour, where the hypertensive female group had a higher mean level compared to the normotensive group (Δ=135.0, and p=0.015 with adjustment for BMI; and Δ=130.9, and p=0.016 without adjustment for BMI).

It is also noted that although the insulin level appears much higher in the female hypertensive group than in the female normotensive group, the difference is not statistically significant due to large standard errors. In our male groups, both leptin, and norepinephrine levels appear much higher in the hypertensive group than in the normotensive group, but the differences again fail to attain statistical significance, due to the same reason (large standard errors), except for the norepinephrine level at 1 hour.

Patient characteristics and leptin, norepinephrine, insulin, and glucose levels during OGTT are summarized in Table 1 and 2 and Figures 1-a to 1-d.

**Figures 1-a:**
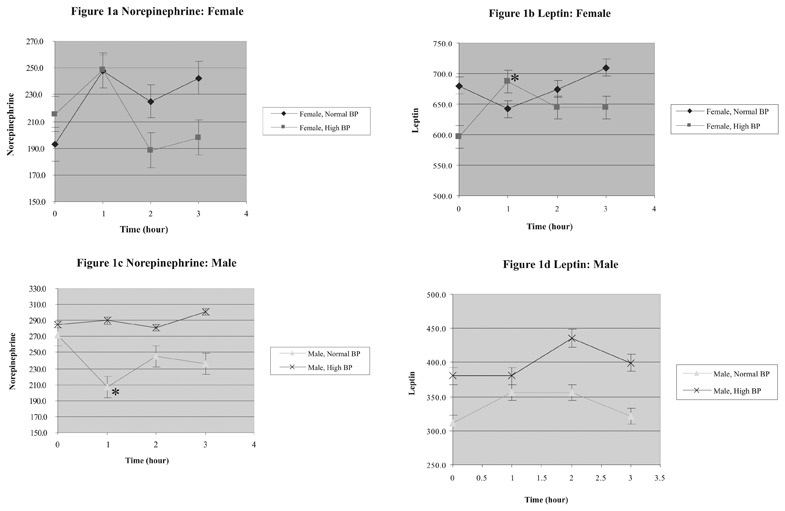
Norepinephrine and Leptin levels during OGTT in the females and males with or without hypertension

**Table 1:**
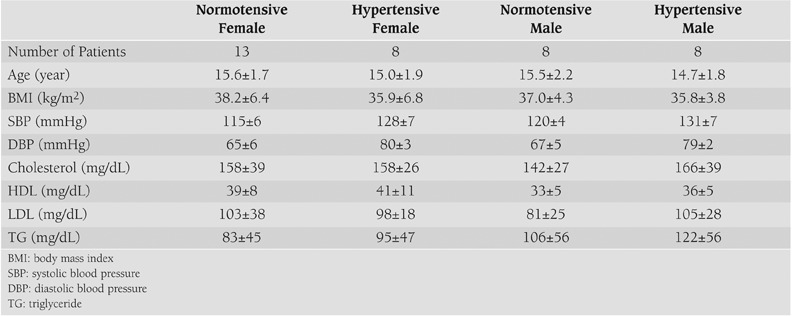
General characteristics of the study groups (Mean±SE)

**2:**
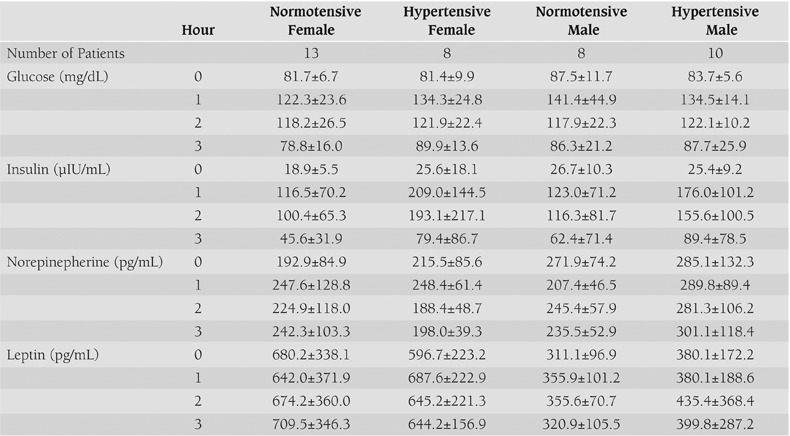
Biochemical data on Glucose, Insulin, Norepinephrine and Leptin levels during OGTT of the study groups (Mean±SE)

## DISCUSSION

Leptin secretion is correlated with BMI and increases during puberty. There is a sexual dimorphism with higher levels in females.^13^ In addition, serum leptin level can change independently of the fat mass. Leptin levels decrease during fasting and increase after meals or glucose loading.^9, 10, 11, 14, 15, 16^

It is postulated that there is a dual interaction of leptin and the sympathetic nervous system. Increasing leptin levels, usually associated with anabolic status such as increased BMI and meals, can suppress satiety associated with weight-reduction and stimulate sympathetic activity which could induce hypertension. Conversely, increased sympathetic nervous system activity suppresses leptin secretion. However, obesity may alter this balance. Based on studies of obese mice, Mark et al^6^ introduced a novel concept of selective leptin resistance which suggests that hyperlepitinemia could contribute to increases in sympathetic activity and increased blood pressure in obese states, where there is a resistance to the metabolic (satiety and weight-reducing) actions of leptin. There are not many clinical studies that were designed to establish a direct relationship between leptin and norepinephrine levels in obesity status. A study by Corica et al^8^ demonstrated a positive association between leptin and norepinephrine changes during OGTT in both normotensive and hypertensive obese women. The authors of that study hypothesized that this association may reflect a lack of leptin suppression by catecholamines or a direct leptin-induced sympathoactivation Recenly, another study by Flanagan et al, “Gender differences in the relationship between leptin, insulin resistance and the autonomic nervous system”,^17^ which included 130 young adults, suggested that the gender difference and leptin in women is associated with sympathetic nervous system activity.

In the present study, the results did not show any significant correlation between leptin and norepinephrine levels in hypertensive or normotensive groups in either gender. However, statistical powers of the tests, which were generally below 0.90, were not sufficient to exclude possible type II errors, due to the small sample size. In the male groups, both leptin and norepinephrine levels were much higher in the hypertensive group. Although the difference did not reach statistical significance of p=0.05 at most time points, we cannot exclude the possibility that this finding could be due to type II errors. Interestingly, the only statistically significant difference between the norepinephrine levels of the two male groups at 1 hour is caused mainly by a decrease of norepinephrine levels at 1 hour in the normotensive group (see Table 2 and Fig. 1-c) instead of an increase in the hypertensive group.

The female groups had higher leptin levels than the two male groups, which is an indication of sexual dimorphism. There was a significant increase in leptin levels at 1 hour in the hypertensive group. This may suggest that sympathetic over-activity manifested by hypertension may be related to hyperleptinemia, which is consistent with the conclusion of a recent study by Flanagan, et al.^17^

Our study represents the first attempt to explore the relationship between leptin, norepinephrine, insulin and glucose levels in obese adolescents. Although no significant relationship between these hormones was observed in this study, our data is still interesting because it reveals different patterns in norepinephrine levels at 1 hour during OGTT for the male groups (a dip for the normotensive group) and in leptin levels at 1 hour during OGTT for the females (an increase for the hypertensive group). These differences imply that norepinephrine and leptin at 0 and 1 hour during routine OGTT for males and females, respectively, may help identify a subgroup of obese adolescents who have higher risks for hypertension and cardiovascular complications.

We realize the limitations of this study: a small sample size, lack of a lean control group, and the method of collection and measurement of norepinephrine levels. Further studies with groups including lean controls, measurement of 24 hour urinary norepinephrine, and larger sample sizes are warranted to confirm our findings.

**Fig. 1-c:**
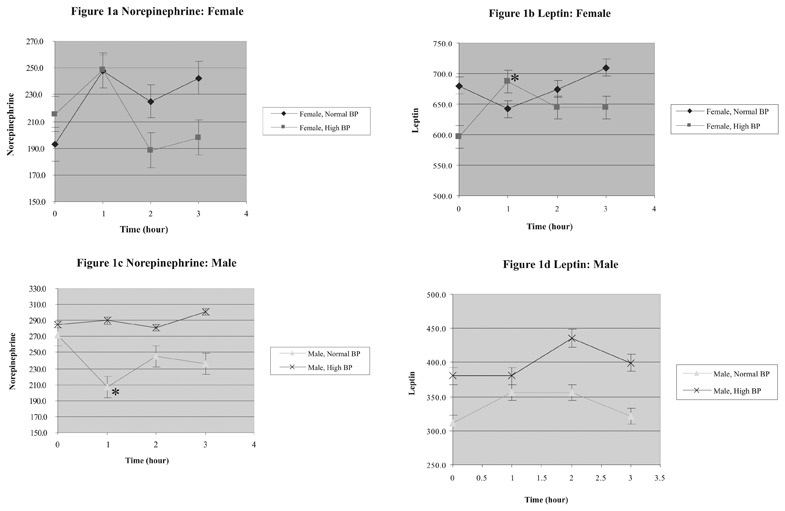
Norepinephrine and Leptin levels during OGTT in the females and males with or without hypertension

**Table 2:**
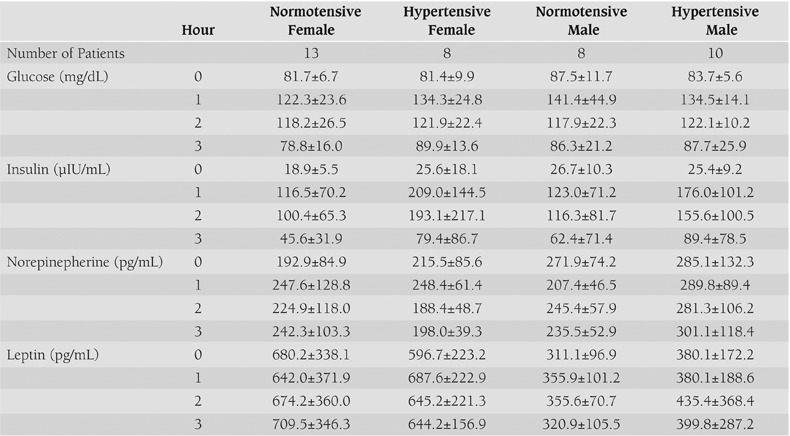
Biochemical data on Glucose, Insulin, Norepinephrine and Leptin levels during OGTT of the study groups (Mean±SE)

## ACKNOWLEDGEMENT

We thank the staff of the CRC and all volunteers who participated in this research. We also thank Dr. Morri Markowitz (Montefiore Medical Center, Albert Einstein College of Medicine) for many helpful suggestions and reviewing this manuscript
